# Nonnulla circa methodum luis Venereæ curandæ meditamenta

**Published:** 1781-10

**Authors:** 


### LONDON MEDICAL JOURNAL,

### For OCTOBER 1781.

### SECTION I.

### BOOKS.

I. *Nonnulla circa methodum luis Venereæ curandæ meditamenta. Auctore* Adolpho Murray, M. D. *et Anatom. Prof.* 4to. Upſal. p. 48.

THIS little work contains a variety of obſervations on the different remedies employed in the cure of venereal complaints. We ſhall ſelect from it ſuch as appear to be the moſt intereſting.

In travelling through Italy, our author was ſurprized to find that in the hoſpitals of that country, inſtead of Mercury, the uſe of which is ſtill prohibited, the phyſicians content themſelves with preſcribing a decoction of the woods, which ſerves only to mitigate the ſymptoms of the diſeaſe, and to emaciate the patients by ſweating.

In a cafe of virulent gonorrhœa Dr. Murray adminiftered the volatile alkali, a remedy ftrongly recommended by M. Peyrilhe, a French writer. The effects it produced were a violent inflammation of the urinary paffages, a fuppreffion of the difcharge, and other alarming fymptoms. Another patient afflicted with venereal ulcers had his complaints greatly exafperated by the ufe of the fame medicine.

Speaking of the different modes of exhibiting mercury, he obferves, that frictions, fumigations, mucilaginous and faline preparations of this mineral have each of them their refpective merits in different circumftances of the difeafe. He confiders the faline as differing from the other preparations of mercury in this refpect only, that the mercury in them is more divided, and for this reafon he thinks they are quicker and more efficacious in their operation.

He advifes us to be cautious in the ufe of fumigations, as the vapour of mercury is apt to excite profufe falivation, or to affect the nervous fyftem, and produce tremor. On this fubject he relates a cafe, communicated to him by Mr. Scopoli, of a man who, upon being expofed to the vapour of fublimate in melting it with copper, fell inftantly into a violent ptyalifm.

Dr.

Dr. Murray obferves, that the faline preparations of mercury frequently occafion fevers, cough, fpafms, and other dangerous complaints, efpecially in perfons fubject to hæmoptoe or the hæmorrhoids. For this reafon he objects to their being given to fuch patients. He is of opinion, that although in general they foon relieve the fymptoms, they hardly ever operate a cure.

He has fometimes experienced very difagreeable effects from the ufe of Keyfer's pills. Of Bellette's fyrup (which confifts of mercury precipitated by an alkali from its folution in the nitrous acid, and mixed with æther and fyrup) he remarks, that it proves efficacious only when given in large dofes, and that it then frequently excites, by its ftimulus, the moft alarming fymptoms.

Dr. Murray difapproves of the mixed method of cure recommended by M. Gardane. He has fometimes found Plenk's remedy of ufe, but he diflikes it becaufe it feparates in the ftomach, and frequently paffes off by ftool.

He prefers the cure by frictions to every other method. He confeffes however that in pregnant women, and in dropfical or phthifical patients, they are not adivfeable; but fimilar objections, he obferves, may be made to every other mer-

 curial curial medicine. He is difpofed to impute the bad effects, which are fometimes found to follow a courfe of frictions, to the fault of the prefcriber or patient, rather than to the remedy.

In fpeaking of frictions he takes occafion to treat very fully of the method practifed at Montpellier, a place much frequented by venereal patients on account of the purity and mildnefs of the climate. Our author is of opinion, however, that in the firft period of the difeafe a very warm air is hurtful. He has obferved, that at Rome and Naples a gonorrhœa is more difficult to cure than the lues itfelf. He therefore afcribes the fuccefs of the Montpellier method to the fuperior excellence of the remedies employed there, rather than to any particular circumftance relative to the climate. This method, which the French writers term *the cure by extinction*, confifts in adminiftering mercurial ointment without exciting a falivation. Bleeding, purging, a light diet, and a frequent repetition of the warm bath, are prefcribed previous to the frictions. Our author is of opinion, that in this way warm bathing promotes the abforption and diftribution of the mercury through the fyftem. For a flight infection he thinks the warm bathing fhould be repeated thirty, and for an an inveterate lues an hundred times. After the ſkin has been ſufficiently prepared by theſe means, the patient is directed to rub in from half a drachm to half an ounce of mercurial ointment every ſecond or third day, till he has conſumed twelve or fifteen ounces of the ointment. If the mouth begins to be affected after ſeven or eight frictions, the ſalivation is to be checked by changing the patient's ſhirt, and by employing ſudorifics, purges, and warm bathing.

The phyſicians at Montpellier have obſerved, that ſcorbutic patients afflicted with the lues can ſeldom be cured of the latter diſeaſe by mercury, unleſs they have been previouſly cured of the ſcurvy. In caſes of venereal phthiſis they preſcribe aſſes and goats milk, lichen iſlandicus, and the juice of ſnails and millepedes previous to the uſe of mercury. If there are any urgent ſymptoms, ſuch as a deep-ſeated caries, an inflamed exoſtoſis, or a dangerous ulcer, they have recourſe immediately to mercury; but as ſoon as the ſymptoms are in a certain degree mitigated, they ſuſpend its uſe till they have gone through the uſual preparatory courſe of bathing, &c. after which they finiſh the cure by frictions.

After deſcribing the Montpellier mode of treatment, our author gives us ſeveral practical remarks.

remarks. If a bubo forms, he thinks the internal ufe of mercury neceffary to the cure. When the glands firft begin to inflame, he recommends the application of extract of faturn and mercurial ointment to the part, with repeated purges to promote a refolution of the tumour. If the bubo fuppurates, he cautions us againft opening it before it is perfectly foft, and then he thinks the knife preferable to a cauftic. He is averfe to our dreffing the wound with mercurials, as the cure, though perhaps more fpeedy, will be fallacious. For the fame reafon he objects to fimilar applications to chancres. In cafes where the fuppuration has not gone on favourably, he has feen good effects from a blifter applied to the gland.

In obftinate exoftofes he advifes us to faw off the difeafed portion of bone, but in cafes of venereal caries he recommends an extract of *Gratiola* or *hedge hyffop*, as a medicine of confiderable efficacy.

